# Coexistence of Pituitary Adenoma and Primary Pituitary Lymphoma: A Case Report and Review of the Literature

**DOI:** 10.3389/fsurg.2022.842830

**Published:** 2022-03-16

**Authors:** Shangjun Ren, Qingyang Lu, Yilei Xiao, Yiming Zhang, Lianqun Zhang, Bin Li, Mengyou Li

**Affiliations:** ^1^Department of Neurosurgery, Liaocheng People's Hospital, Liaocheng, China; ^2^Department of Pathology, Liaocheng People's Hospital, Liaocheng, China; ^3^Department of Neurosurgery, Beijing Neurosurgical Institute, Capital Medical University, Beijing, China

**Keywords:** collision tumor, pituitary adenoma (PA), pituitary lymphoma, DLBCL - diffuse large B cell lymphoma, review

## Abstract

In the pituitary sella, the coexistence of pituitary adenoma and primary pituitary lymphoma is exceedingly rare. Thus far, only six cases have been reported. Here, we present the seventh case of coexisting pituitary adenoma and primary pituitary lymphoma, which was difficult to differentiate from other sellar tumors. To our knowledge, this is the first case of the prolactin subtype of the pituitary adenoma in literature. We have also systematically reviewed the literature and summarized the characteristics of coexisting pituitary adenoma and lymphoma. We believe this report provides a new clinical reference for the diagnosis and treatment of collision tumors of pituitary adenoma and lymphoma.

## Introduction

Collision tumor in the sellar region is a rare condition; it refers to two coexisting tumors of different shapes attached to each other. Typically, a collision tumor in the sellar region is characterized by the coexistence of pituitary adenoma and another tumor. Reportedly, the pituitary adenoma can be associated with meningioma (1), Rathke's cleft cyst (2), craniopharyngioma (3), gangliocytoma (4), and other neoplasms. Among them, the coexistence of pituitary adenoma and primary pituitary lymphoma is extremely rare. It is difficult to distinguish it from other sellar region tumors, and the perioperative diagnosis is difficult because the clinical and radiological manifestations of most cases are similar to pituitary adenomas. Only six cases of pituitary adenoma associated with lymphoma have, thus far, been reported. Here, we report the seventh case and systematically review the literature.

## Case Presentation

**Figure 1:**
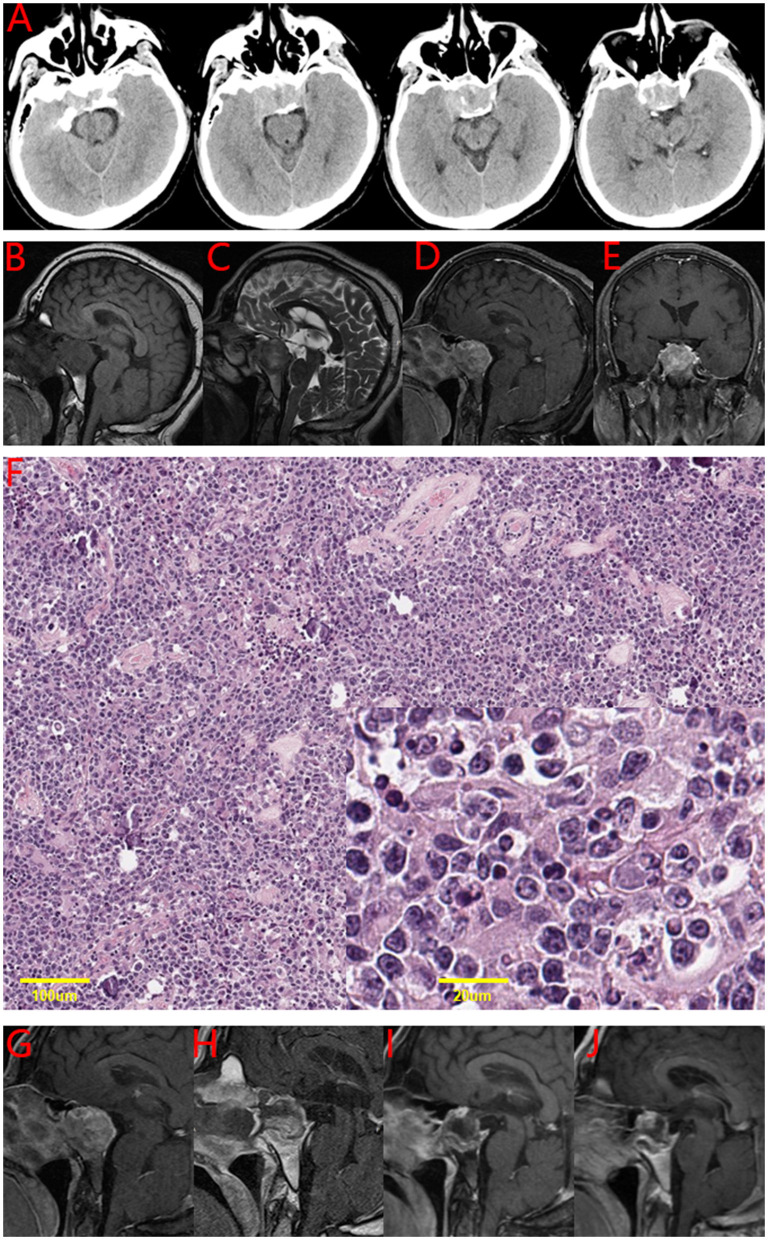
The imaging and pathological results of the patient. **(A)** Brain CT of the patient in this case. Brain CT showed a tumor in the sellar region with calcification. **(B-E)** Brain MRI of the patient in this case. **(B)** T1-weighted image: sellar region tumor is imaged in the same intensity as the cerebral cortex. **(C)** T2-weighted image: a slightly longer signal. **(D)** and **(E)** post-gadolinium image: significant enhancement of under-homogeneity. **(F)** Hematoxylin and eosin stain of the tumor tissue in this case. Primary pituitary diffuse large B-cell lymphoma combined with sparsely granulated lactotroph adenoma. **(G-J)** A series of MRIs of the patient in this case. **(G)** Preoperative **(H)**, postoperative **(I)**, 6 months after the operation **(J)**, and 12 months after the operation.

In May 2020, a 41-year-old Asian male was hospitalized with a history of intermittent stabbing headache, without progressive deterioration of visual acuity or visual field. Table 1 presents the results of the endocrine examination. Brain CT showed a tumor with calcification in the sellar region (Figure 1A). The brain MRI showed that the sellar floor was eroded; further, the pituitary stalk was not clear. The tumor showed hypointensity in both T1-weighted and T2-weighted MRI images. A contrast-enhanced MRI showed a significant enhancement of under-homogeneity (Figures 1B–E). These radiological manifestations indicated that the sellar mass was pituitary adenoma.

**Table 1:** Endocrine examination results.

**Hormone**	**Level measured**	**Normal levels**
GH	0.51 ng/ml	0.014-5.219 ng/ml
PRL	11 ng/ml	3.7-17.9 ng/ml
ACTH	**94.05 pg/ml**	10.1-57.6 pg/ml
TSH	**6.84 mIU/L**	0.465-4.680 mIU/L

*Bold text indicates abnormal data*.

*GH, growth hormone; PRL, prolactin, ACTH, adrenocorticotropic hormone; TSH, thyroid-stimulating hormone*.

The patient underwent endoscopic endonasal transsphenoidal tumor resection. A tough tumor was encountered in the sphenoid sinus and sellar region. The tumor was abnormally hypervascular, and there was bone inside the tumor. The volume of intraoperative bleeding was about 200 mL. By the end of the surgery, all the tumors were removed in pieces. Postoperatively, the patient recovered well. The symptoms of headache were significantly relieved. Pathological results showed sparsely granulated lactotroph adenoma combined with primary pituitary diffuse large B-cell lymphoma (Figure 1F). The immunohistochemistry results are shown in Table 2.

**Table 2:** Immunohistochemistry results.

**Biomarker**	**Result**
CD45, CD20, CD79α, CD10, Bcl-6, c-MYC, MUM1, Pit-1, PRL, Syn, CK18	Positive (+)
Bcl-2, SF-1, T-Pit, GH, TSH, FSH, LH, ACTH	Negative [-]

A postoperative head MRI showed that there was no residual tumor in the sellar region. A PET-CT scan did not show increased uptake in the pituitary gland and other parts. The results of bone marrow puncture showed that the degree of proliferation was V/VII, G:E = 2.3:1, and nuclear heterogeneous cells were occasionally seen. Then, he received four cycles of chemotherapy. The R-MI protocol (rituximab+methotrexate+ifosfamide) was used, accompanied by supportive treatment.

The patient underwent follow-up examinations at postoperative 6 months and 1 year. The patient was generally in good condition, and the tumor did not recur. A series of brain MRIs showed no evidence of tumor regrowth (Figures 1G–J). The patient will continue to be followed up.

## Discussion

Collision tumors of pituitary adenoma and primary pituitary lymphoma in the sellar region are extremely rare. We systematically reviewed the literature to identify the characteristics of coexisting pituitary adenoma and lymphoma. To our knowledge, thus far, only seven cases have been reported, including this case. We have summarized the characteristics of these seven cases (5–10) and presented them in Table 3.

**Table 3:** Summary cases of coexistence of pituitary adenoma and pituitary lymphoma.

**Case report**	**Age/Sex**	**Symptoms**	**Treatment**	**Subtype of PA**	**Subtype of PL**	**Recurrence**
Kuhn. (5)	67/F	VD, VFD	ST	FSH	T-LBL	Yes (6 Mo)
Au. (6)	82/M	HA, VD, VFD	BIOP, RT	TSH	DLBCL	ND
Romeike. (7)	64/F	HA, VFD	ST, MT, RT	FSH	T-LBL	No (19 Mo)
Martinez. (8)	71/F	VFD	ST, RT	GH	DLBCL	No (12 Mo)
Ban. (9)	74/M	HA	ST, MT	FSH	DLBCL	No (32 Mo)
Gupta. (10)	55/F	HA, VD	ST	ACTH	T-LBL	ND
Present case	41/M	HA	ST, MT	PRL	DLBCL	No (12 Mo)

*PA, pituitary adenoma; PL, pituitary lymphoma; F, female; M, male; VD, visual deterioration; VFD, visual field defect; HA, headache; ST, surgical treatment; BIOP, biopsy; RT, radiation therapy; MT, medical treatment; FSH, follicle-stimulating hormone; TSH, thyroid-stimulating hormone; GH, growth hormone; PRL, prolactin; T-LBL, T-cell lymphoblastic lymphoma; DLBCL, diffuse large B-cell lymphoma; Mo, month; ND, not described*.

The pathophysiological mechanism of tumorigenesis remains unclear, as there are only a very small number of cases currently reported. The local immune changes in the pituitary gland may be brought about by tumorigenesis. It is reported that primary pituitary lymphoma can develop in immunosuppressed individuals (11). At the same time, the occurrence and development of pituitary adenomas are closely related to immune infiltration (12). Whether immune-related mechanisms are involved in the occurrence of collision tumors is still poorly understood and needs to be investigated in more patients.

The coexistence of pituitary adenoma and primary pituitary lymphoma has no special clinical manifestations. The most common clinical manifestation is headache; it was noted in 5 of 7 cases (Table 3). Whether the patient's vision and visual field are affected mainly depends on the size of the tumor. Eyesight decline or visual field defect in patients occurred in five cases, but not in the present report (Table 3). Imaging findings are similar to pituitary adenomas, which makes preoperative diagnosis very challenging (Figure 1). Pathology is the gold standard for diagnosis. The diagnosis of collision tumor of pituitary adenoma and primary pituitary lymphoma in all seven cases was confirmed by pathology. The subtypes of pituitary adenoma can be follicle-stimulating hormone (FSH), Thyroid-stimulating hormone (TSH), Growth hormone (GH), Adrenocorticotropic hormone (ACTH), or Prolactin (PRL). The FSH subtype of pituitary adenoma is the most common (Table 3). This case report was the first to suggest the PRL subtype of pituitary adenoma. The subtypes of pituitary lymphoma are also not consistent. It can be T-cell lymphoblastic lymphoma (T-LBL) or diffuse large B-cell lymphoma (DLBCL). It is different from primary pituitary lymphoma. The most common subtypes of primary pituitary lymphoma are B-cell lymphoma, followed by T cell and NK/T cell types (13).

Surgical management is the most important treatment option for collision tumors of pituitary adenoma and primary pituitary lymphoma. The patients in the six previous cases underwent surgical treatment. In Au et al.'s (6) case report, the patient was an 82-year-old man. Perhaps because of advanced age, the patient did not undergo surgical resection after tumor biopsy but underwent radiotherapy. With the development of neuroendoscopic technology, endoscopic transnasal resection of collision tumors in the sellar region has become the treatment of choice. In our case, the patient underwent endoscopic endonasal transsphenoidal tumor resection. In the end, all the tumors were removed in pieces. If the tumor is completely resected and there is no evidence of systemic lymphoma, no additional radiotherapy is required. However, DLBCL is a highly malignant lesion. To prevent tumor recurrence, chemotherapy can be used appropriately. If the tumor remains or there is evidence of systemic lymphoma, additional radiotherapy or chemotherapy is required. Chemotherapy was used in three cases (Table 3). The classic chemotherapy drug is methotrexate. The external beam radiation of the head was used in three cases (6–8). In the case report by Romeike et al. (7), we found that radiotherapy and chemotherapy were used after the subtotal removal of the lesion. A thorough postoperative examination, including brain MRI, PET-CT, bone marrow aspirate, and bone marrow trephine biopsy, are necessary to reveal any sign of systemic manifestation of the lymphoma spread.

The recurrence rate after total resection is low, but there is still recurrence. In the case report by Kuhn et al. published in 1999 (5), the tumor recurred 6 months after the operation. Perhaps, the available surgical technology then was not optimal, failing to completely remove the tumor in the first operation. Moreover, the patient did not receive chemotherapy and radiotherapy after the first operation. The patient, therefore, had to undergo a second operation. The control of collision tumors is more difficult than simple pituitary adenoma, so the follow-up cycle needs to be prolonged. We suggest that the follow-up should be extended to more than 3 years after the initial operation. If the tumor relapses, a reoperation is still the first choice, but radiotherapy and chemotherapy need to be considered after the operation.

## Conclusions

The coexistence of pituitary adenoma and primary pituitary lymphoma in the sellar region is extremely rare. The disease diagnosis, tumor control, and overall survival are more challenging to achieve for collision tumors than for pituitary adenomas alone. The gross total resection of both tumors is very important for satisfactory tumor control. The follow-up assessment of patients should be appropriately prolonged.

## Limitation

Because the patient's preoperative diagnosis was a conventional pituitary adenoma, video recording was not performed during the operation. Therefore, there are no intraoperative images. Furthermore, the report lacks neuro-ophthalmological examination, because the patient claimed to have normal visual acuity and field. Lastly, only 1-year postoperative follow-up was performed on the patient. We plan to continue to monitor the situation of this patient.

## Data Availability Statement

The raw data supporting the conclusions of this article will be made available by the authors, without undue reservation.

## Ethics Statement

Written informed consent was obtained from the individual(s) for the publication of any potentially identifiable images or data included in this article.

## Author Contributions

ML conceived the study and edited the final manuscript. SR and QL collected the clinical data and performed a literature review. YZ confirmed the pathological analysis. BL reviewed the clinical notes and produced the draft manuscript. YX and LZ conceived the research and helped with the writing of the manuscript. All authors read and approved the final manuscript.

## Funding

This work was supported by grants from the Taishan Scholar Project of Shandong Province of China (tsqn202103200) and the National Natural Science Foundation of China (81701159).

## Conflict of Interest

The authors declare that the research was conducted in the absence of any commercial or financial relationships that could be construed as a potential conflict of interest.

## Publisher's Note

All claims expressed in this article are solely those of the authors and do not necessarily represent those of their affiliated organizations, or those of the publisher, the editors and the reviewers. Any product that may be evaluated in this article, or claim that may be made by its manufacturer, is not guaranteed or endorsed by the publisher.

## Abbreviations

CT: Computed tomography
MRI: Magnetic resonance imaging
FSH: Follicle-stimulating hormone
TSH: Thyroid-stimulating hormone
GH: Growth hormone
PRL: Prolactin
T-LBL: T-cell lymphoblastic lymphoma
DLBCL: Diffuse large B-cell lymphoma
PET: Positron emission tomography.
